# GLP-1R Signaling and Functional Molecules in Incretin Therapy

**DOI:** 10.3390/molecules28020751

**Published:** 2023-01-11

**Authors:** Wenwei Wan, Qikai Qin, Linshan Xie, Hanqing Zhang, Fan Wu, Raymond C. Stevens, Yan Liu

**Affiliations:** 1 iHuman Institute, ShanghaiTech University, School of Life Science and Technology, ShanghaiTech University, Shanghai 201210, China; 2 iHuman Institute, ShanghaiTech University, Shanghai 201210, China; 3 Structure Therapeutics, South San Francisco, CA 94080, USA

**Keywords:** GLP-1R signaling, functional molecules, incretin therapy, drug discovery, type 2 diabetes mellitus

## Abstract

Glucagon-like peptide-1 receptor (GLP-1R) is a critical therapeutic target for type 2 diabetes mellitus (T2DM). The GLP-1R cellular signaling mechanism relevant to insulin secretion and blood glucose regulation has been extensively studied. Numerous drugs targeting GLP-1R have entered clinical treatment. However, novel functional molecules with reduced side effects and enhanced therapeutic efficacy are still in high demand. In this review, we summarize the basis of GLP-1R cellular signaling, and how it is involved in the treatment of T2DM. We review the functional molecules of incretin therapy in various stages of clinical trials. We also outline the current strategies and emerging techniques that are furthering the development of novel therapeutic drugs for T2DM and other metabolic diseases.

## 1. Introduction

Diabetes is considered the fastest-growing global health problem, affecting about 10% of adults around the world [1,2]. The diabetic population will reach 783 million globally in 2045 according to an estimate from the International Diabetes Federation [2]. All types of diabetes share the common clinical manifestation of hyperglycemia and several characteristic symptoms, including thirst, polyuria, constant hunger, fatigue, weight loss, and blurred vision [2]. As the disease progresses, complications such as retinopathy, nephropathy, and neuropathy may occur, and the risk of cardiovascular diseases, obesity, and nonalcoholic fatty liver disease will increase, which significantly affects the quality of life.

T2DM accounts for more than 90% of diabetes cases [1,2], although this percentage might be higher since approximately one-third of people living with T2DM are undiagnosed [2]. Adopting a healthy lifestyle and taking metformin are the cornerstone for T2DM management. A combination of sulfonylureas, alpha-glucosidase inhibitors, thiazolidinediones, and insulin injections can be added when the single antidiabetic medication is insufficient. However, these drugs can cause side effects such as hypoglycemia, weight gain, and cardiovascular risk [3].

Pancreatic β-cell dysfunction and resultant insulin deficiency are the key features of T2DM [4], however, most medications do not target β-cell and become less effective as diabetes progress [5]. Fortunately, a couple of gut-derived natural peptides termed incretins that can stimulate insulin secretion [6] have inspired novel T2DM treatments. Incretins were first discovered upon observing that oral glucose administration leads to greater insulinotropic effects than intravenous administration [7]. Since then, researchers began to investigate gut-derived insulin secretagogues; and incretin-based therapies currently have become the preferred first injection therapy for T2DM treatment, due to their strong glycemic control effect and remarkable safety profile [8]. Incretins include glucagon-like peptide-1 (GLP-1) and glucose-dependent insulinotropic polypeptide (GIP). Although both GLP-1 and GIP could promote insulin secretion in a healthy state, the therapeutic potential of GIP alone is controversial [9,10]. In contrast, the GLP-1 receptor (GLP-1R) is thought to be one of the most important potential drug targets for glucose-dependent T2DM treatment, lowering hypoglycemia risk compared to insulin and sulfonylureas [11].

GLP-1, as the endogenous agonist of GLP-1R [12,13,14], is a promising natural antidiabetic product due to its anorexigenic, insulinotropic, and weight-reducing effects [15]. However, in vivo GLP-1 can be cleaved by dipeptidyl peptidase 4 (DPP-4) immediately after secretion at the second amino acid (alanine) from its N-terminal [16]. This will lead to an instant degradation of GLP-1 and a short circulation time in the human body. Although GLP-1 has many potential advantages, its short circulation time of about 2 min [17] limits its application in treatment since such frequent administration is incompatible with patient compliance and thereby reducing drug effectiveness.

DPP-4 inhibitors and GLP-1 analogs with prolonged circulation time have already been applied in incretin therapy and have performed well. However, GLP-1R agonists are favored because they have superior body weight control [18] and cardiovascular outcomes [19]. In this review, we discuss the current GLP-1R signaling and ligand development strategies, trends in incretin therapy, and perspectives on T2DM treatment.

## 2. GLP-1R Signaling 

### 2.1. The Structural Basis of GLP-1R

**Figure 1:**
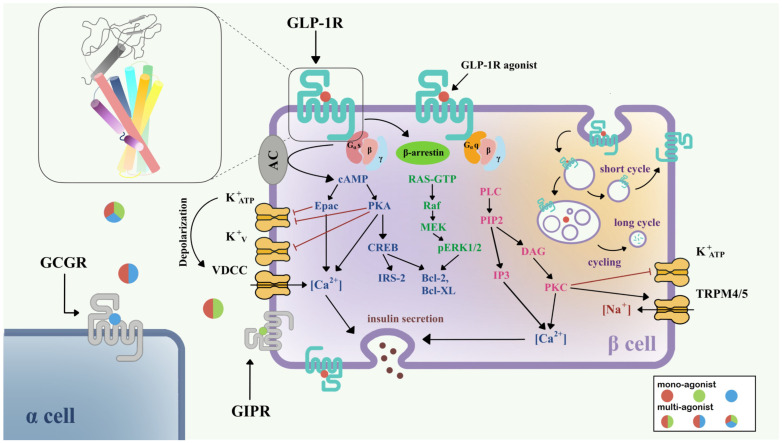
Signaling pathways of GLP-1R in pancreatic β-cell [15,30]. Three downstream signaling pathways initiated from Gαs (blue), Gαq (pink), and β-arrestin (green) are shown in the left half of the β-cell. The GLP-1R internalization process and insulin secretion after GLP-1R activation are also shown. Colored circles show mono-agonists targeting GLP-1R (red), GIPR (green), and GCGR (blue), and multi-agonists targeting GLP-1R/GIPR (red/green), GLP-1R/GCGR (red/blue), and GLP-1R/GIPR/GCGR (red/green/blue). The top left inset shows the structure of GLP-1R with a seven-transmembrane helix bundle and a large ECD. Abbreviations: VDCC, voltage-dependent calcium channel; TRPM, the transient receptor potential melastatin; GCGR, glucagon receptor; GIPR, gastric inhibitory polypeptide receptor.

G protein-coupled receptors (GPCRs) are widely distributed in various tissues and play key roles in a diversity of physiological activities. As the largest receptor family, GPCRs are important drug targets for a broad range of indications [20]. GPCRs share a conserved seven-transmembrane helix bundle (Figure 1) with three extracellular loops (ECLs) and three intracellular loops (ICLs). The ECLs form an extracellular surface that interacts with orthosteric ligands. While the ICLs, to large extent, determine downstream receptor signaling. GLP-1R, together with four other glucagon receptors (GCGR, GLP-2R, GIPR, and GHRHR), belongs to the secretin (class B1) GPCR family, whose endogenous ligands are peptide hormones [21,22]. Class B1 GPCRs have a large and structurally conserved extracellular domain (ECD) of 120–160 residues at the N-terminal, forming a three-layered α-β-β-α fold that is stabilized by three interlayer disulfide bonds [23].

The endogenous ligands of GLP-1R are GLP-1 (7-36) and GLP-1 (7-37), products from the post-translational processing of proglucagon [24]. Proglucagon also produces several other peptide hormones for receptors in the glucagon receptor family, such as glucagon, oxyntomodulin (OXM), and GLP-2. On binding with endogenous peptides, the glucagon receptor family shares a similar recognition mode, which is described as a “two-domain” binding mode. The C-terminal α helix of peptide ligand initiates peptide recognition by binding to the ECD, then the peptide N-terminal can activate the receptor and trigger its downstream signaling cascade by binding to the transmembrane domain (TMD) ligand-binding pocket [25]. Recently released cryo-EM structures of GLP-1R in complex with a peptide ligand revealed that peptides form a single helix in binding post, which is a unique feature shared in class B1 GPCRs [26,27,28].

### 2.2. Signaling Pathways of GLP-1R

Researchers have been pursuing functional studies of GLP-1R for many years to illuminate the mechanism of GLP-1R signaling. GLP-1R downstream signaling pathways network can be activated through coupling with the intracellular transducers [29]. The diverse protein-binding forms will lead to complex downstream pathways.

Currently, it is believed that GLP-1R predominantly signals through the Gα_s_/cAMP pathway; however, there is evidence that GLP-1R couples with Gα_q_ and other G proteins. After activation, GLP-1R undergoes phosphorylation at the C-terminal, which further recruits β-arrestin, leading to internalization and desensitization of the receptor (Figure 1). The Gα_s_/cAMP pathway directly leads to the glucose-induced secretion of insulin granules [31,32]. After activation by full agonists such as GLP-1, GLP-1R couples with Gα_s_, activates adenylate cyclase (AC), and causes the accumulation of cAMP [33]. With increasing cAMP levels, protein kinase A (PKA) [31] and the exchange protein directly activated by cAMP-2 (Epac-2) [34] are also activated. PKA and Epac-2 trigger the closure of K_ATP_ and K_V_ channels, which depolarizes the cell membrane, opens voltage-dependent calcium channels (VDCC), and causes Ca^2+^ influx [35,36]. In addition to the classical function of cAMP, the cAMP/CREB pathway could also induce the expression of insulin receptor substrate 2 (IRS2) and promote β-cell survival, demonstrating the protective effects of GLP-1 analogs on β-cells [37].

In addition to the Gα_s_/cAMP pathway, GLP-1R is also able to couple with other G protein subtypes including Gα_i_, Gα_q_, Gα_o_, and Gα_11_ [38,39]. Recent research has demonstrated the importance of the Gα_q_ pathway in the pancreatic β-cell. First, the Gα_q_ pathway initiates from the coupling of phospholipase-C (PLC), transforming phosphatidylinositol 4,5-bisphosphate (PIP2) to inositol triphosphate 3 (IP3) and diacylglycerol (DAG), and accompanies the activation of protein kinase C (PKC) and intracellular Ca^2+^ influx production via IP3 receptor [39]. PKC also triggers the closure of the K_ATP_ channel and activation of TRPM4/5 [40]. Gα_s_ and Gα_q_ regulate the cAMP and Ca^2+^ levels, respectively; however, variational mechanisms exist in the complex network of G protein signaling pathways. A switch of Gα_s_ and Gα_q_ pathways has been found under certain conditions, including persistent depolarization of cell membrane [30], and reduction in GLP-1 concentration to picomolar [41]. The switch of G protein pathways clarifies a possible mechanism for basal insulin secretion under drug treatment and provides important direction for incretin therapy in T2DM.

Two types of β-arrestin, β-arrestin-1 and β-arrestin-2, mediate the GLP-1R downstream signaling in β-cells, and also elicit the receptor internalization process. β-arrestin-1 mediates the phosphorylation of CREB and ERK1/2 [42], further phosphorylating Bad (Bcl-xL/Bcl-2-associated death promoter homolog) and inhibiting cell apoptosis [43]. β-arrestin-2 functions as an indispensable insulin regulator. Knocking out β-arrestin-2 in the mice model and leading to impaired insulin secretion [44]. With the treatment of sulfonylureas, direct interaction between β-arrestin-1 and Epac-2 can upregulate the Ca^2+^ concentration [45]. 

GLP-1R is a fast-internalized and recycling receptor [46]. After activation, GLP-1R-ligand complexes enter the endosome [32]. A portion of that is eventually transported to lysosomes for degradation, while the other portion returns to the cell membrane [46]. Different agonists may show different effects on GLP-1R internalization and recycling. For example, GLP-1 is apt to receptor recycling, while exendin-4 may favor slower recycling and lysosome targeting [47]. The latest study suggested that internalized endosomal GPCR-Gα_s_ complexes are the origin of induced ERK activity [48], but the detailed mechanism remains implicit.

Due to advantageous physiological effects and rapid response in GLP-1R signaling pathways, functional molecules targeting GLP-1R are attracting increasing attention in drug discovery. Optimization of the current molecules and discovery of novel compounds are highly demanded.

## 3. Functional Molecules Targeting GLP-1R

### 3.1. Peptide-Based Mono-Agonists

**Figure 2:**
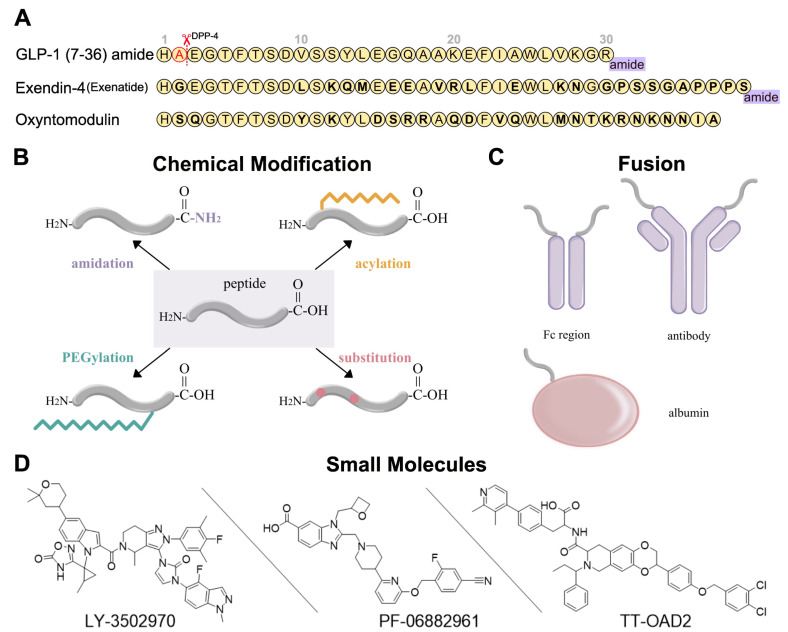
Development strategies for GLP-1R ligands [16,52,53]. (**A**) Amino acid sequences of GLP-1 (7-36) amide, exendin-4, and oxyntomodulin. Different residues from GLP-1 are in bold type. DPP-4 deactivates the GLP-1 (7-36) amide by cleaving it after the second amino acid (alanine) from the N-terminal and leads to the short circulation time of GLP-1 (7-36) in human body. (**B**) Several chemical modifications and their combinations can be applied in GLP-1R peptide agonist development, including C-terminal amidation, acylation, PEGylation, and substitution. (**C**) GLP-1R peptide ligands can fuse to Fc region of antibody, antibody, and albumin. (**D**) Three typical small-molecule agonists for GLP-1R: LY-3502970 and PF-06882961 are under clinical trials, while TT-OAD2 has failed.

The short circulation time of endogenous GLP-1R agonists, such as GLP-1, limits its potential for T2DM treatment. Fortunately, exendin-4, a 39-amino acid peptide from Gila monster (*Heloderma suspectum*) venom, showed similar characteristics to the mammalian incretin hormone [49,50]. It naturally has better resistance to DPP-4 cleavage due to its different amino acid sequence (Figure 2A), and thus has a much longer circulation time than GLP-1. Eventually, exendin-4 was developed as the first marketed twice-daily GLP-1R peptide agonist, exenatide [51]. Although exenatide represents a huge step forward, antidiabetic drugs with even longer circulation times for better patient compliance are still in demand. 

Peptide optimization strategies for GLP-1R peptide agonists include sequence alteration, chemical modification, and fusion. The most obvious approaches to resist degradation are sequence alteration (Figure 2A) and amino acids substitution with α-amino iso-butyric acid (Aib) at the cleavage site [54,55,56] (Figure 2B). As for chemical modification, C-terminal amidation, PEGylation, fatty acid acylation, and amino acid substitution are the main methods to improve peptide stability in vivo (Figure 2B). C-terminal amidation can not only limit proteolytic degradation, thus extending the circulation time of the peptide, but also improve the binding affinity with the receptor [57]. PEGylation attaches polyethylene glycol (PEG) units to the peptide [58], while fatty acid acylation allows the covalent attachment of a fatty acid chain on the peptide [59] (Figure 2B). PEGylation and fatty acid acylation can increase the molecular mass of peptides, improving resistance to renal filtration and enzymatic degradation due to steric hindrance [53,60]. Beyond that, fatty-acid acylated peptides can spontaneously oligomerize at the subcutaneous injection site and delay absorption due to their amphiphilicity, resulting in a sustained-release effect [61,62,63]. The fatty acid chain can also protect the peptide monomer by reversibly binding to albumin [53]. These strategies are usually undertaken in conjunction with each other to generate a better protective effect. 

In addition to chemical modifications, we can also fuse the peptide to a long-circulating and low immunogenic protein, such as an antibody fragment (Fc region), antibody, and albumin (Figure 2C). This strategy can prolong the peptide half-life in two ways. First, like PEGylation and fatty acid acylation, the increased molecular mass can improve resistance to enzymatic degradation and renal filtration. Second, the fusion protein can go through the neonatal Fc receptor (FcRn) recycling pathway [64].

**Table 1:** Peptide-based mono-agonists targeting GLP-1R.

Name	Trade Name	Administration	Indications	Status ^1^	Ref.
Exenatide	Byetta	SC, bid	T2DM	2005	[51,65,66]
	Bydureon	SC, qw	T2DM	2012	[67]
Liraglutide	Victoza Saxenda	SC, qd	T2DM Obesity	2010 2014	[68,69]
					[70]
Dulaglutide	Trulicity	SC, qw	T2DM	2014	[71]
Albiglutide	Tanzeum	SC, qw	T2DM	2014 (withdrawal 2018)	[72,73]
Lixisenatide	Adlyxin	SC, qd	T2DM	2016	[74]
Beinaglutide	Yishengtai	SC, tid	T2DM	2016	[75]
Semaglutide	Ozempic Rybelsus Wegovy	SC, qw PO ^2^, qd SC, qw	T2DM T2DM Obesity	2017 2019 2021	[76]
					[77]
					[78]
PEG-Loxenatide	Fulaimei	SC, qw	T2DM	2019	[79]
PEGylated Exenatide (PB-119)	/	SC, qw	T2DM	Phase 3	[80]
Efpeglenatide (SAR439977)	/	SC, qw	T2DM	Phase 3	[81,82]
Vurolenatide	/	SC, bim	SBS	Phase 2	/
JY09	/	SC, qw	T2DM	Phase 2	/

^1^ Year of approval, or the final phase reached. So far, all approved GLP-1R agonists are derived from peptides such as GLP-1 or exendin-4. ^2^ Rybelsus is the only approved oral peptide-based drug. Abbreviations: SC, Subcutaneous injection; bid, twice daily; qw, once weekly; qd, once daily; PO, oral administration; bim, twice monthly; T2DM, type 2 diabetes mellitus; SBS, short bowel syndrome. Data were manually collected from clinicaltrials.gov and Drugs@FDA database on 15 December 2022.

### 3.2. Peptide-Based Multi-Agnosits

Rather than single agonists targeting only GLP-1R, dual or triple agonists targeting additional receptors (GIPR, GCGR, and GLP-2R) involved in the incretin axis and/or other pathways (Table 2) are expected to have stronger glucoregulatory, weight-reducing, and even cardiovascular protective effects [4]. Although the mechanism by which multi-agonists have superior effects is unknown, many clinical results have shown their therapeutic potential.

Thus far, GIPR/GLP-1R is the most attractive target combination. Instead of playing a redundant role, GIP may protect β-cells from dysfunction and destruction independently of GLP-1 [83]. Although the therapeutic potential of GIPR agonists is debatable, clinical trials of dual GIPR/GLP-1R agonists have produced promising results. The combination of these two GPCR pathways is assumed to increase glucose-dependent insulin secretion, decrease energy consumption, improve white adipose tissue function, and increase insulin sensitivity [84]. Tirzepatide, the first dual agonist for the treatment of T2DM and obesity targeting GIPR and GLP-1R, was approved by the U.S. FDA in 2022. Compared with existing drugs such as semaglutide, once-weekly tirzepatide showed better results in glycated hemoglobin A1c (HbA1c) [85] and body weight reduction [86] with a similar safety profile.

GCGR/GLP-1R dual agonists have also received much attention. OXM can naturally activate both GCGR and GLP-1R with lower potency than their primary ligands [87]. Although glucagon and GLP-1 have distinct effects on glucose levels, their effects on food intake may be additive [88], leading to more significant body-weight reduction. Several OXM derivatives are under clinical trials, for example, cotadutide, a once-daily GCGR/GLP-1R dual agonist, has shown promising impacts on glycemic control, body weight, and liver fat reduction [89].

**Table 2:** Dual/triple-agonists targeting GLP-1R and other GPCRs.

Name	Trade Name	Administration	Indications	Status ^1^	Ref.
**GLP-1R/GIPR**					
Tirzepatide	Mounjaro /	SC, qw	T2DM Obesity	2022 2022 FTD ^2^	[90,91] [86]
CT-868	/	SC, qd	T2DM	Phase 1	/
NNC0090-2746 (RG7697)	/	SC, qd	T2DM	Phase 2	[92]
**GLP-1R/GLP-2R**					
Dapiglutide (ZP7570)	/	SC, qw	SBS	Phase 1	/
**GLP-1R/GCGR**					
SAR425899	/	SC, qd	T2DM	Phase 2 (discontinued)	[93]
Pemvidutide (ALT-801)	/	SC, qw	Obesity NASH	Phase 1 Phase 1	/ /
Pegapamodutide (LY2944876)	/	SC, qw	T2DM	Phase 2 (discontinued)	/
Cotadutide (MEDI0382)	/	SC, qd	T2DM NASH CKD Obesity	Phase 2 Phase 2 Phase 2 Phase 1	[94] [95] [96] [97]
Efinopegdutide (MK-6024)	/	SC, qw	NASH	Phase 2	/
Mazdutide (IBI-362)	/	SC, qw	T2DM Obesity	Phase 2 Phase 1	/ [98]
BI456906	/	SC, qw	T2DM Obesity	Phase 2 Phase 2	/ /
MK-8521	/	SC, qd	T2DM	Phase 2	/
PB-718	/	SC, qw	NASH	Phase 1	/
NN9277 (NNC 9204 1177)	/	SC, qw	Obesity	Phase 1 (discontinued)	/
MOD6031	/	SC, qw	Obesity	Phase 1	/
**GLP-1R/GCGR/GIPR**					
HM-15211	/	SC, qw	NAFLD	Phase 1	/
LY-3437943	/	SC, qw	T2DM	Phase 1	[99]

^1^ Year of approval, the final phase reached. ^2^ The U.S. FDA granted fast track designation (FTD) to Tirzepatide in October 2022. Among these drug candidates, only tirzepatide has been approved. Abbreviations: SC, subcutaneous injection; bid, twice daily; qw, once weekly; qd, once daily; T2DM, type 2 diabetes mellitus; SBS, short bowel syndrome; CKD, chronic kidney disease; NASH, non-alcoholic steatohepatitis; NAFLD, non-alcoholic fatty liver disease. Data were manually collected from clinicaltrials.gov and Drugs@FDA database on 15 December 2022.

Clinical trial failures may occur in some cases of dual or triple agonists, caused by their side effects. In order to develop safer therapeutic drugs, it is essential to study the mechanism of these dual and triple agonists [88] from structural, functional, and pharmacologic aspects.

### 3.3. Small Molecule Agonists and Positive Allosteric Modulators (PAM)

So far, all marketed GLP-1R agonists are peptide-based and were developed from natural products such as GLP-1 and exendin-4 (Table 1). Although their half-lives can be prolonged by the strategies discussed above, problems or limits such as cost, side effects, and subcutaneous injection remain [100]. In the expectation of improving these deficiencies, many groups and major pharmaceutical companies have long been pursuing the development of non-peptide drugs. Due to a poor understanding of the ligand binding mode and activation mechanism prior to the first GLP-1R structure being solved in 2017 [101], high-throughput screening was adapted in many studies to identify promising candidates, followed by massive structure–activity relationship (SAR) studies to improve the chemical and pharmacokinetic properties of compounds.

**Table 3:** Small molecule agonists and PAM targeting GLP-1R.

Name	Frequency	Indications	Status ^1^	Ref.
Danuglipron (PF-06882961)	bid	T2DM obesity	Phase 2 Phase 2	[102]
TTP-273	qd/bid	T2DM	Phase 2	/
LY3502970 (OWL833)	qd	T2DM obesity	Phase 2 Phase 1	[103]
PF-07081532	qd	T2DM	Phase 1	[104]
RGT-075	qd	T2DM	Phase 1	[105]
TT-OAD2	/	/	Preclinical (discontinued)	[106]

^1^ Year of approval, or the final phase reached. All these small molecules are designed for oral administration. Abbreviations: bid, twice daily; qd, once daily; T2DM, type 2 diabetes mellitus. Data were manually collected from clinicaltrials.gov on 15 December 2022.

Currently, none of the small molecule GLP-1R agonists have been approved. However, several candidates are under clinical trial (Table 3). There are fewer small molecule agonists and PAMs targeting GLP-1R than peptide-based drug candidates, and most of them are still in the early stage of development. One of the first small-molecule agonists, Boc5, is a substituted cyclobutane identified by HT screening [107]. However, it has not been launched into clinical study. PF-06882961, based on diazabenzimidazoles, is a full agonist in cAMP elevation, but a partial agonist in other signaling pathways [108,109]. TTP-273, which has completed phase 2 trials, is an azoanthracene derivative reported in several patents [110,111]. Another compound in the same series as TTP-273, TT-OAD2, is a partial agonist with slow kinetics in promoting cAMP [106]. LY3502970 is a pyrazolopyridine derivative and is a biased agonist that abolished β-arrestin signaling [103,112]. The chemical structures of PF-07081532 and RGT-075 have not yet been disclosed.

**Figure 3:**
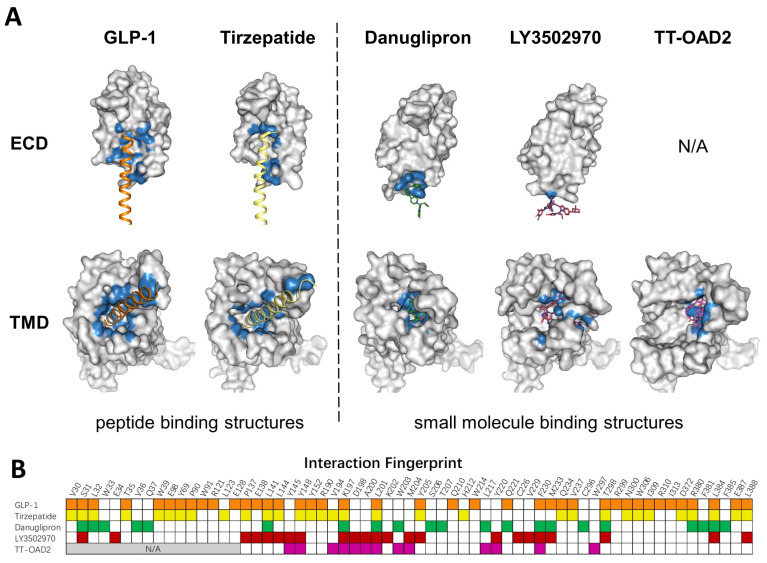
Comparison of peptide and small molecule binding pockets. (**A**) Structures of GLP1R in complex with GLP1 (PDB: 6X18), tirzepatide (PDB: 7RGP), danuglipron (PDB: 6X1A), LY3502970 (PDB: 7E14), and TT-OAD2 (PDB: 6ORV) are shown in the separated ECD and TMD. GLP-1R is shown as a gray surface model. Residues within 4 Å of the corresponding ligand are colored in blue. (**B**) Specific residues that interact with corresponding ligands in a fingerprint array. Ligands in (A) and their interaction fingerprints in (B) appear as follows: GLP-1 (orange), tirzepatide (yellow), danuglipron (green), LY3502970 (red), TT-OAD2 (magenta). N/A, not applicable. ECD was not resolved in TT-OAD2 binding structure.

Several GLP-1R structures in complex with different small molecules have been released later, and revealed dramatic diversity in the binding mode of each compound (Figure 3A). TT-OAD2 forms a U-shape conformation in the GLP-1R helical bundle near the ECD where it interacts with residues within the transmembrane helix 1 (TM1) to TM3 and ECLs [106]. LY3502970 sits in a similar position as TT-OAD2, while both its arms insert into slits between helixes, clamping around TM2. PF06882961 stays in the orthosteric binding pocket in the TMD, almost overlapping with the binding sites of the N-terminal of GLP-1 [28]. Additionally, both PF06882961 and LY3502970 have vital π–π interactions with W33 in the ECD, which abolished their effects on GLP-1R in rodents [103]. Finally, a Boc5 binding structure is published recently, which shows that Boc5 adapts a claw shape in its binding pocket, with three fingers stuck into the slits between TM1-TM3 and TM7 [113].

Many allosteric modulators have also been reported in the past few years. As one of the first reported small molecules, compound 2 was extensively studied over the years and was characterized as an ago-PAM [114,115], a type of molecule that possesses functions of both agonist and PAM. Another set of ago-PAMs based on pyrimidines represented by BETP was identified by screening a small library generated through a pharmacophore model [116]. Recently, a new ago-PAM, LSN3318839, was reported to restore the activity of GLP-1 (9-39) [117]. However, none of these allosteric modulators has entered clinical study.

To mimic peptide interaction and to obtain sufficient affinity when occupying the peptide pocket, most small molecules end up having a relatively large molecular weight, which could affect the physical properties and pharmacokinetics of compounds. Though most PAMs or ago-PAMs have lower molecular weight, none of them has entered clinical trial. As revealed by the structures, TT-OAD2, LY3502970, and PF-06882961 have quite different binding pockets: only three residues interact with all three compounds, 13 residues interact with two out of three, and 22 residues interact with only one of them (Figure 3B). Different binding modes of each compound may contribute to differences in efficacy and biased agonism. As peptide-based drug development has entered the era of dual agonists, a clearer understanding of the activation and biased signaling mechanism of GLP-1R is needed to aid the design of ideal compounds. 

## 4. Incretin Therapeutic Studies

### 4.1. GLP-1R Agonists Based T2DM Therapy in Clinical Trials

In the early stage of diabetes, metformin monotherapy is widely applied as the first-line drug and is generally sufficient for glycemic control in T2DM [118]. With the progression of the disease, metformin alone cannot maintain blood glucose at the desired level, and combination therapies become necessary. The selection of combined medication, including sulfonylureas, alpha-glucosidase inhibitors, thiazolidinediones, dipeptidyl peptidase 4 inhibitors (DPP4i), GLP-1 agonists, and sodium-dependent glucose transporters 2 inhibitors (SGLT2i) [2,119], is based on clinical characteristics and patients’ preferences. 

Among these drugs, thiazolidinediones and sulfonylureas are commonly used and cost-effective. However, they can cause congestive heart failure and hypoglycemia, respectively, thus limiting their further application. GLP-1R agonists and SGLT2i both have fewer side effects and are recommended for patients with comorbidities. Meta-analysis showed that the combination of a GLP-1R agonist and metformin could effectively reduce blood glucose levels [120]. Insulin injection is inevitable for glycemic control after the dysfunction of non-insulin treatment.

Among the non-insulin medication, incretin therapy is an advanced treatment for T2DM patients. The therapeutic potential of incretin in T2DM has been explored for more than one hundred years. In 1906, Moore et al. used extracts from the duodenal mucous membrane to treat T2DM [121]. Half a century later, Nauck et al. discovered an impaired incretin response in T2DM patients, leading to a decrease in incretin-stimulated insulin release [122]. A further study identified that the reduction in GLP-1 accounted for the diabetic state [6]. A 6-week pilot study investigated the long-term effect of GLP-1 in T2DM by continuous administration of this peptide hormone. Patients had lower fasting glucose (4.3 mmol/L) and HbA1c (−1.3%) under the administration of GLP-1 [123]. However, due to the short circulation time of native GLP-1, it is infeasible to continuously administrate native GLP-1 for long-term blood glucose management [124]. Some GLP-1 analogs were developed to overcome this problem. 

Exenatide, the first approved GLP-1 analog, has significant effects on HbA1c level reduction [125], weight loss, and glycemic control [125], but no cardioprotective effect [67]. As the plasma drug concentration could be maintained for 12 h, the method of administration was by subcutaneous injection twice a day. Liraglutide was another novel GLP-1 analog that was approved by the FDA in 2010. A series of phase 3 trials (Liraglutide Effect and Action in Diabetes, LEAD) was launched to compare the efficacy and safety between liraglutide and other oral antidiabetic drugs [69,126,127]. In the LEAD-3 study, a significant reduction in HbA1c level was found with a dosage of 1.8 mg liraglutide compared with glimepiride monotherapy [126]. Moreover, compared with exenatide, liraglutide carries a lower risk of cardiovascular death [120]. Throughout the LEAD trials, patients showed high tolerance to the drug, with the most frequently reported side effects being GI events such as nausea and vomiting. Liraglutide was found to maintain a maximum plasma drug concentration for about 12–14 h, so the period of administration became once daily. Most recently, the GLP-1 analogs have continuously improved, representing great advances in treating T2DM, obesity, and cardiovascular diseases. Semaglutide Unabated Sustainability in Treatment of Type 2 Diabetes (SUSTAIN) trials showed the positive effects of subcutaneous semaglutide on glycemic control [128]. Weekly 2.4 mg semaglutide, compared with daily 3 mg liraglutide, shows a superior effect on weight loss [129]. Oral semaglutide administered once per week showed favorable efficacy and safety in the treatment of T2DM patients and the large, randomized control clinical trial is ongoing [130]. 

In addition to GLP-1R agonists, the discovery of multi-agonists is also ongoing in recent years. The first approved dual agonist, tirzepatide, is a potent agonist for both GLP-1R and GIPR. At dosages of 5, 10, and 15 mg, tirzepatide showed non-inferior treatment in glycemic control and superior behavior in weight loss than 1 mg semaglutide [85], further demonstrating the therapeutic efficacy of multi-agonists. Many other multi-agonists have emerged and are in the process of early-stage clinical trials (Table 2). 

### 4.2. Adverse Effects of Current Available GLP-1R Agonists

Although GLP-1R agonists possess multiple beneficial effects and are a promising next-generation therapy for T2DM, their application still has many contraindications. Due to initial concerns about potential risks in preclinical studies [8], they are not recommended for T2DM patients with diseases such as severe gastrointestinal disease, medullary carcinoma of the thyroid [131], multiple endocrine neoplasm syndrome type 2, and history of pancreatitis [126]. Specifically, the following adverse effect profiles of GLP-1R agonists should be taken into consideration when formulating a clinical treatment plan. Firstly, among all the adverse effects of GLP-1R agonist treatment, nausea and related gastrointestinal responses, such as vomiting and diarrhea, are the most common [132,133]. These unwanted gastrointestinal responses reduce patient compliance and are important reasons for discontinuing T2DM treatment [134,135]. Secondly, the roles of GLP-1R agonists in promoting the proliferation of β-cells and reducing their apoptosis could increase the risk of pancreatic cancer [136,137]. Further, several concerns remain about the correlation of GLP1R agonist treatment with many other adverse events, including eye disorders such as retinopathy [138,139,140], and gallbladder- or biliary-related events [141]. In the case of Efpeglenatide, retinal safety, severe gastrointestinal events, and kidney function have attracted sustained attention [82]. Although not all the above adverse effects related to GLP-1R agonists treatment have been supported by recent evidence [142,143], further research and rigorous clinical trials examining the incidence of these events are still needed.

### 4.3. Strategies for Optimizing GLP-1R Agonists

Despite the numerous anti-hyperglycemic agents that are already available on the market for T2DM treatment, there are still a surprising number of drug candidates in clinical trials, of which no less than 40% are with novel therapeutic molecules or targets [144,145]. With this rapid development of clinical therapeutic solutions for treating diabetes, a burst of potential first-in-class drugs is expected that would further reduce adverse effects while maintaining or even enhancing the efficacy of current drugs. Such advances would provide more choices for diabetes management and would be beneficial to personalize medicine with better patient compliance.

Many strategies have been conducted to further optimize GLP-1R agonists. For example, in the quest for longer-lasting peptides, a series of GLP-1R G-protein biased agonists have been developed via backbone modifications with normal or enhanced G-protein signaling, but significantly reduced β-arrestin response, leading to the improved duration of action of the peptides [133]. Compared with “nonbiased” agonists, these biased GLP-1 agonists would lead to reduced receptor desensitization, prolonged availability of GLP-1R at the cell surface, enhanced insulinotropic and glucose-lowering properties, and greater therapeutic value. Among all these agents, replacing the first eight amino acids of exendin-4 (HGEGTFTS) with novel sequence ELVDNAVGG, the modified agent named P5, was a potent and selective biased GLP-1 agonist that was investigated, which was found to have a higher acute anti-hyperglycemia efficacy than exendin-4 [135]. A recent study reported that PX17, an updated agent based on P5, shows greater blood glucose modulation and body weight reduction compared with semaglutide [146]. Exendin-phe1, with minimal substitutions within the N-terminus of exendin-4, was found to promote glycemic benefits without decreasing tolerability [147]. Structure and functional studies, aided by molecular dynamics simulation, illustrate that the peptide agonist conformation plasticity, especially the dynamic interaction of the receptor with the N-terminal activation domain of the peptide, may be an essential determinant for agonist efficacy [148]. Thus, biased GLP-1R agonists generated via backbone modification provide a powerful strategy for improving therapeutic efficacy and have led to novel treatments for T2DM.

Recent studies have reported strategies, which altered the brain penetration property of GLP-1R agonists, to mitigate nausea or other unwanted gastrointestinal responses, the most prominent adverse effects of GLP-1R agonist treatment [134,149]. The adverse and glucose-lowing effects probably possess at least partially different signaling response profiles. Thus, researchers could rationally design for separating these two signaling pathways to tune down the unwanted adverse effects via directed modifications. Nausea, or other unwanted gastrointestinal responses, may be triggered by the effects of GLP1R agonists targeting the receptors expressed in the central nervous system (CNS). Blood glucose regulation effects mainly depend on the targeting of the pancreas or other peripheral systems. One possible strategy would be to downregulate the effects of GLP1R agonists on the CNS. Since all the agents gain access to the brain by penetrating the blood–brain barrier, this protection mechanism could be utilized as a key step to filter out unwanted agents. Studies utilizing the corrination method, conjugating GLP1R agonists with vitamin B12 or related compounds containing corrin ring, affect pharmacokinetics and modify the solubility of GLP1R agonists and prevent them from penetrating into the CNS. Specifically, covalent conjugation of extendin-4 to vitamin B12, which possesses a corrin ring structure, forms B12-exendin-4 with reduced brain penetrance, leading to glucose lowering while significantly abolishing the unwanted emetic events [134]. Conjugation of extendin-4 with Dicyanocoinamide, the B12 precursor, could also exhibit similar glucoregulatory capability and nausea reduction effect [149]. These approaches have effectively reduced emetic effects, while retaining efficient glycemic control.

## 5. Conclusion and Perspective

In summary, the importance of incretin in blood glucose modulation has been fully recognized [119]. Incretin therapy is the focus of significant drug development and has become one of the most promising therapies for T2DM. GLP-1R agonists, as the most prominent glucose-lowering agents in incretin therapy, possess unique advantages in T2DM treatment, with low hypoglycemic risk, clear cardioprotective effects, superior body weight loss, and other associated clinical benefits [8]. 

Currently, all marketed GLP-1R agonists are derived from natural peptides, including GLP-1, OXM, and exendin-4. Several strategies, such as chemical modification, antibody or albumin fusion, multi-agonist design, etc., have been applied to improve the efficacy and pharmacokinetics of GLP-1R agonists. However, the demand for novel agents with better therapeutic efficacy and attenuated adverse effects remains strong. 

The complexity of downstream GLP-1R signaling pathways determines both the antidiabetic and potential adverse effects of GLP-1R agonists. A comprehensive understanding of GLP-1R structure and its functional pathways is critical for novel drug/therapy development for T2DM treatment. The increasing availability of high-quality GLP-1R structures in different states can help us decipher the factors that alter cell signaling, receptor trafficking, and biased agonism. This insight is essential for the rational design of drugs that can selectively activate certain signaling pathways. Notably, combinational computational approaches may accelerate this process. Furthermore, novel screening technology, such as affinity selective mass spectrometry (ASMS), could promote the discovery of lead compounds from natural products that act as reservoirs of novel therapeutic agents [29,150,151]. 

As a chronic complex metabolic disease, T2DM requires long-term management. However, the inconvenience of injection administration as well as the difficulties of managing multiple drugs for treating complications can lead to poor adherence. Novel oral GLP-1R agonists can overcome this problem, to a certain extent, and we are glad to see many small-molecule oral drug candidates under clinical trials. With transcellular permeation enhancers, peptide-based GLP-1R agonists may also be taken orally [152]. Moreover, polypill (fixed-low-dose combination drug) can also help by simplifying medication [153,154,155,156] and, if oral antidiabetic drugs were included in the polypill formulation, diabetic patients would further benefit [157,158]. 

It is critical to implement incretin therapy before severe pancreatic β-cell mass destruction or dysfunction. Unfortunately, in recent decades, the population of adolescents and young adults with T2DM is increasing [159], which highlights the urgency of early diagnosis. Although conventional diabetes diagnostic indicators such as fasting plasma glucose and HbA1c are simple and practicable, they do not provide the specificity to distinguish pathways related to pancreatic β-cell mass destruction or dysfunction [160,161]. Noninvasive imaging tools (such as PET and MRI) and novel biomarkers can provide abundant pathological characteristics about β-cells [162,163,164,165,166] or related diagnostic information [140,167,168,169,170,171,172,173], which may help improve monitoring disease progress and severity, support the development of diabetes management strategies, evaluate and even guide drug development [174,175]. Artificial intelligence (AI) is likely to play an increasingly important role in diabetes diagnosis (such as medical image analysis and subtype classification), clinical decision support, management, risk identification, prevention [133,134,135,136], and prognosis [176]. These applications in incretin therapy could bring better treatment efficacy. 

The significance of incretin therapy based on GLP-1R and its future potential has been clearly shown. While successful advances have been made in solving structures, developing/optimizing drugs, and decoding downstream signaling pathways of GLP-1R, integration of the progress on novel GLP-1R agonists development, early diagnosis, treatment, management, prevention, and personalized medicine will ultimately lead to the resolution of T2DM globally.

## Data Availability

The data presented in this study are available on request from the corresponding author.
